# Hyperoxia-induced deterioration of diastolic function in anaesthetised patients with coronary artery disease – Randomised crossover trial

**DOI:** 10.1016/j.bjao.2023.100135

**Published:** 2023-04-27

**Authors:** Jan O. Friess, Jan Mikasi, Rico Baumann, Rajevan Ranjan, Kady Fischer, Anja Levis, Sandra Terbeck, Trevor Hirschi, Daniel Gerber, Gabor Erdoes, Florian S. Schoenhoff, Thierry P. Carrel, Raouf Madhkour, Balthasar Eberle, Dominik P. Guensch

**Affiliations:** 1 Department of Anaesthesiology and Pain Medicine, Inselspital, Bern University Hospital, University of Bern, Bern, Switzerland; 2 Department of Anesthesiology, Critical Care and Pain Medicine, Boston Children's Hospital, Harvard Medical School, Boston, MA, USA; 3 Department of Cardiovascular Surgery, Inselspital, Bern University Hospital, University of Bern, Bern, Switzerland; 4 Department of Cardiology, Inselspital, Bern University Hospital, University of Bern, Bern, Switzerland

**Keywords:** coronary artery disease, diastolic function, general anaesthesia, hyperoxia, myocardial function, normoxaemia, perioperative ischaemia

## Abstract

**Background:**

There are no current recommendations for oxygen titration in patients with stable coronary artery disease. This study investigates the effect of iatrogenic hyperoxia on cardiac function in patients with coronary artery disease undergoing general anaesthesia.

**Methods:**

Patients scheduled for elective coronary artery bypass graft surgery were prospectively recruited into this randomised crossover clinical trial. All patients were exposed to inspired oxygen fractions of 0.3 (normoxaemia) and 0.8 (hyperoxia) in randomised order. A transoesophageal echocardiographic imaging protocol was performed during each exposure. Primary analysis investigated changes in 3D peak strain, whereas secondary analyses investigated other systolic and diastolic responses.

**Results:**

There was no statistical difference in systolic function between normoxaemia and hyperoxia. However, the response in systolic function to hyperoxia was dependent on ventricular function at normoxaemia. Patients with a normoxaemic left ventricular (LV) global longitudinal strain (GLS) poorer than the derived cut-off (>–15.4%) improved with hyperoxia (*P*<0.01), whereas in patients with normoxaemic LV-GLS <–15.4%, LV-GLS worsened with transition to hyperoxia (*P*<0.01). The same was seen for right ventricular GLS with a cut-off at –24.1%. Diastolic function worsened during hyperoxia indicated by a significant increase of averaged *E*/*e*′ (8.6 [2.6]. *vs* 8.2 [2.4], *P*=0.01) and *E*/*A* ratio (1.4 (0.4) *vs* 1.3 (0.4), *P*=0.01).

**Conclusions:**

Although the response of biventricular systolic variables is dependent on systolic function at normoxaemia, diastolic function consistently worsens under hyperoxia. In coronary artery disease, intraoperative strain analysis may offer guidance for oxygen titration.

**Clinical trial registration:**

NCT04424433.

Approximately 30% of patients who undergo major surgery have cardiovascular disease and consequently have a higher perioperative risk for cardiac complications.^1^, ^2^, ^3^, ^4^ During general anaesthesia numerous drug effects and perturbations of physiological variables may impact perioperative outcome. One of these factors may be supplemental oxygen, which is routinely administered at varying dosage and duration. It is recommended practice to use a high fraction of inspired oxygen (*F*io_2_) for preoxygenation during critical periods of general anaesthesia, for example induction und airway management, in order to prevent deoxygenation.^5^ For maintenance of general anaesthesia, international anaesthesia societies do not provide uniform recommendations on indications and dosage of delivered oxygen concentration. A recent survey revealed substantial variability in utilisation of supplemental oxygen, with *F*io_2_ ranging from normoxaemic to hyperoxic levels.^6^ Potential links between iatrogenic hyperoxia during general anaesthesia and perioperative, particularly cardiac, outcomes came into focus recently.^7^

Hyperoxia is known to be associated with coronary vasoconstriction. Hyperoxia-induced reduction of coronary blood flow may lead to ischaemic events in susceptible myocardium.^8^, ^9^, ^10^ Therefore, current guidelines for treating non-dyspnoeic patients with myocardial ischaemia stipulate to refrain from giving supplemental oxygen as long as oxygen saturation (*S*po_2_) is more than 90% or arterial partial pressure of oxygen (*P*ao_2_) is greater than 60 mm Hg (8 kPa).^11^, ^12^, ^13^ Recent work demonstrated that hyperoxia caused myocardial function in a subset of awake patients with stable chronic coronary artery disease.^14^ It is not known if these findings translate to the perioperative setting as the myocardial effects of iatrogenic hyperoxia during general anaesthesia still a matter of debate.

Assessment of systolic and diastolic function using transoesophageal echocardiography (TOE) has become routine during cardiac surgery. Recently, myocardial deformation (strain) analysis has been shown to be a sensitive perioperative marker of systolic function.^15^, ^16^, ^17^

The objective of this study was to investigate the effects of hyperoxia in comparison with normoxaemia on biventricular function assessed by TOE in anaesthetised patients before coronary artery bypass graft (CABG) surgery. The primary goal was to investigate changes in 3D myocardial peak strain as a marker of systolic function between targeted *F*io_2_ levels. Secondary analyses investigated changes in traditional echocardiographic variables including diastolic function and ejection fraction, and if echocardiographic findings at normoxaemia contributed to individual responses to hyperoxia.

## Methods

### Study design

The Myocardial Strain Analysis in Anaesthetized Coronary Artery Disease Patients During Hyperoxia and Normoxaemia (StrECHO-O_2_) trial is an investigator-initiated, single-centre, randomised cross-over trial with partially blinded echocardiographic image data analysis. The study was conducted at Inselspital, Bern University Hospital, Switzerland, and was approved by the Cantonal Research Ethics Board of Bern (2020–00145, April 2020), registered on clinicaltrials.gov (NCT04424433) and on the Swiss National Clinical Trials Portal (SNCTP000003911). Patients were enrolled after written informed consent was obtained. The trial was performed in accordance with the Declaration of Helsinki. The detailed trial design has been published.^18^

### Population

Patients scheduled for elective CABG surgery were enrolled prospectively (*n*=109, June 2020 to June 2021; Supplementary Figure S1). These patients had well-defined coronary artery disease and underwent a perioperative TOE examination as part of the clinical anaesthesia protocol. All patients received general anaesthesia and intraoperative TOE examination. Exclusion criteria were emergency surgery, acute ischaemia, atrial fibrillation, and severe-grade valvular disease (Supplementary methods).^18^

### Intervention

After standardised induction of general anaesthesia and before surgical incision, patients were consecutively exposed to either a period of normoxaemia, with an *F*io_2_ of 0.3 followed by hyperoxia with an *F*io_2_ of 0.8, or *vice versa* according to the cross-over randomisation schedule with equal allocation per group. If required during normoxaemia, *F*io_2_ was further adjusted to target a *S*po_2_ between 95% and 98%. After 5 min of steady-state controlled mechanical ventilation, the oxygen status was confirmed via arterial blood gas analysis, and the pre-defined TOE images were obtained for each *F*io_2_ level for the assessment of changes in cardiac function.

### Cardiac imaging

All TOE images and cine loops were acquired using Philips EPIQ 7c (X7-2t probe) or EPIQ CVx (X8-2t probe, EPIQ Ultrasound System; Philips Medical Systems, Andover, MA, USA). During the study period under general anaesthesia, TOE examinations were performed by the anaesthetists who had targeted the *F*io_2_ levels and exclusively by European Association of Cardiovascular Imaging (EACVI)-certified echocardiographers. As previously described, after establishing a stable respiratory and haemodynamic equilibrium, standard two-dimensional (2D), 3D, M-mode, and Doppler images were acquired to assess systolic and diastolic function, both at normoxaemia and hyperoxia.^18^ Immediately after image acquisition, the following measurements were made by the echocardiographer: tricuspid annular plane systolic excursion (TAPSE), septal and lateral tissue Doppler velocities, *E* (early) and *A* (atrial) transmitral inflow velocities derived by pulse waved Doppler (PW), and left ventricular outflow tract velocity time integral. The 3D images were then coded, and the remaining analyses were performed by blinded readers offline, using TOMTEC-Arena (TOMTEC Imaging Systems, Unterschleissheim, Germany). This included quantification of 3D left and right ventricular volumes, ejection fraction (LVEF and RVEF), RV fractional area change (FAC) and 3D strain. The key variables acquired from the strain analysis were global peak longitudinal strain of the left ventricle (LV-GLS) and of the right ventricular free-wall (RV-GLS), whereas other measures of strain including, end-systolic strain, time to peak strain, and mechanical dispersion were also derived. The workflow is described in further detail in Supplementary methods.

### Outcomes

The primary outcome was the difference in 3D longitudinal global peak strain measured in 3D TOE datasets, obtained in the same individual at normoxaemia and hyperoxia. Secondary outcomes were other volumetric and functional variables derived from 3D LV and RV assessment, and standard echocardiographic descriptors of systolic and diastolic function. Anaesthesia and haemodynamic variables were recorded and patient characteristics.

### Statistical analysis

For the comparison of the two *F*io_2_ levels, a mixed-effects general linear model was used that included the randomised group category as a cofactor to account for possible order effect. The difference (Δ) in each variable between the oxygenation levels was then calculated (*F*io_2_ 0.8–*F*io_2_ 0.3) to determine if individuals worsened or improved with hyperoxia. We considered the normoxaemic state as our arbitrary baseline of myocardial function, as it is closest to the patients' status at room air, though with the clear limitation of disregarding other effects of general anaesthesia.

The ability of normoxaemic (*F*io_2_=0.3) imaging results to discriminate the individual echocardiographic variable response to hyperoxia was assessed using receiver operating characteristic (ROC) curves and the associated area under the curve (AUC). The binary outcome for this ROC analysis was defined as either worsening or improving in the functional marker with the transition from normoxaemia to hyperoxia. For peak strain and diastolic variables, a worsening was defined as an increase (Δ>0) of the respective variable. For volumetric markers and TAPSE, a worsening was defined as a decrease (Δ<0). Ultimately to derive the ROC curve, the true positive and false positive rates were examined for varying thresholds of the normoxaemia measurements (detailed in Supplementary methods). From the generated ROC curves, an optimal cut-off point was derived using the Youden index, a value which represents the best compromise between sensitivity and specificity. Additional thresholds to designate individuals with a high probability of improving or worsening of the respective measurements with hyperoxia were defined based on the 95% sensitivity and specificity of the ROC curves. For interobserver reliability, a second blinded reader analysed 20 random datasets for the primary endpoint (LV-GLS) and the diastolic variables, then an intra-class correlation (ICC) was calculated for absolute agreement. Statistical significance was defined with a two-sided *P*-value of <0.05. GraphPad Prism version 9 (GraphPad Software Inc., La Jolla, CA, USA), and IBM SPSS Statistics 26 (IBM Corp., Armonk, NY, USA) were used for analysis.

## Results

### Patients

Patient enrolment is detailed in the Consolidated Standards of Reporting Trials (CONSORT) diagram (Supplementary Figure S1). Of the 152 patients screened for eligibility, 109 provided informed consent and were included. Six patients were subsequently excluded during the study for medical or technical reasons: difficult TOE probe placement (*n*=3), new-onset atrial fibrillation (*n*=2), or failure to reach the targeted degree of hyperoxia (*n*=1). The patient characteristics were similar in both groups (Table 1). Standard 2D intraoperative measures were assessed in all patients for both *F*io_2_ levels (*n*=206 datasets). For 3D LV analysis, 176 of 206 (85%) of the 3D LV images and 180 of 206 (87%) 3D RV images could be successfully analysed with 82 of 103 (80%) patients having usable 3D datasets for both exposures (Supplementary Figure S1).

**Table 1:** Patient characteristics. Frequency (percent), mean (standard deviation) or (range) for age specifically are displayed for the entire group, and split by the order of the first *F*io_2_ level. ACE, angiotensin-converting enzyme; H, hyperoxia first; LAD, left anterior descending; LCx, left circumflex; N, normoxaemia first; RCA, right coronary artery.

	All patients (*n*=103)	N group (*n*=54)	H group (*n*=49)
*Baseline*			
Sex (males), *n* (%)	90 (87)	47 (87)	43 (88)
Age (yr)	66 (45–81)	67 (49–79)	65 (45–81)
Height (cm)	173 (8)	174 (8)	172 (9)
Weight (kg)	87 (16)	88 (18)	84 (13)
BMI (kg m^−2^)	28.9 (4.7)	29.3 (5.5)	28.4 (3.7)
*Medical history, n* (%)			
Dyslipidaemia	39 (38)	20 (37)	19 (39)
Diabetes mellitus	36 (35)	22 (41)	14 (29)
Hypertensive heart disease	15 (15)	8 (15)	7 (14)
Obesity (BMI >30 kg m^−2^)	27 (26)	15 (28)	12 (25)
Sleep apnoea	10 (10)	8 (15)	2 (4)
Heart failure	4 (4)	2 (4)	2 (4)
*Medications, n* (%)			
Statins or fibrates	85 (83)	46 (85)	39 (80)
Anti-platelets	83 (81)	43 (80)	40 (82)
Beta blockers	68 (66)	38 (70)	30 (61)
ACE inhibitors	30 (29)	13 (24)	17 (35)
Angiotensin II receptor blockers	25 (24)	14 (26)	11 (22)
Calcium channel blocker	21 (20)	13 (24)	8 (16)
Diuretics	20 (19)	9 (17)	11 (22)
Anticoagulants	14 (14)	6 (11)	8 (16)
Dual anti-platelets	12 (12)	9 (17)	3 (6)
Other	41 (40)	24 (44)	17 (35)
*Number of vessels affected, n* (%)			
1	8 (8)	5 (9)	3 (6)
2	34 (33)	18 (33)	16 (33)
3	61 (59)	31 (57)	30 (61)
*Vessel affected, n* (%)			
LAD	100 (97)	52 (96)	48 (98)
LCx	86 (84)	46 (85)	40 (82)
RCA	73 (71)	36 (67)	37 (76)

### Global myocardial function

**Fig 1:**
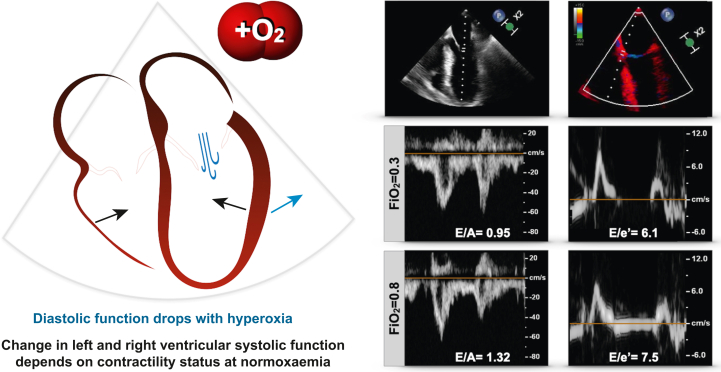
Diastolic function deteriorates with hyperoxia. Two-dimensional echocardiography measurements of transmitral flow (centre) and tissue Doppler imaging (right) significantly worsen when the fraction of inspired oxygen (*F*io_2_) is 0.8 in comparison with normoxaemia (*F*io_2_=0.3).

There was no statistical difference in LV-GLS between normoxaemia and hyperoxia. Nor was there a significant difference between RV-GLS, ventricular volumes, ejection fraction, and cardiac output, as obtained from 3D volumetry (Supplementary Table S1). Diastolic dysfunction was present at both oxygen levels, demonstrated by septal (<8 cm s^−1^) and lateral (<10 cm s^−1^) tissue velocities below accepted cut-offs (Table 2). The transmitral inflow peak *E* velocity was significantly accelerated at hyperoxia, whereas peak *A* velocity was reduced (*E*: 62 [15] *vs* 65 [16] cm s^−1^, *P*=0.04; *A*: 50 [17] *vs A*: 48 [15] cm s^−1^, *P*=0.04). Consequently, *E*/*A* and *E*/*e*′ ratios were significantly increased during the hyperoxic state (Table 2, Fig 1), indicating worsened diastolic function. For all variables, the randomly allocated order of O_2_ exposure had no significant statistical impact. A good reliability (ICC=0.80, *P*<0.01) was found between readers for the primary outcome measurement of LV-GLS. Furthermore, an excellent reliability (ICC≥0.94, *P*<0.01) was shown for the diastolic function variables (Supplementary Table S2).

**Table 2:** Intraoperative measures. Mean (standard deviation) or median [min–max] are displayed for measurements obtained at each level. For all variables, there was no significance in the order that the levels were performed. Additional volumetric and circumferential strain parameters are provided in Supplementary Table S1. ∗*P*<0.05 between gas levels. *F*io_2_, inspired oxygen fraction; GLS, global longitudinal peak strain; LV, left ventricle, LVOT; VTI, left ventricular outflow tract velocity time integral; ORi, oxygen reserve index; *P*aco_2_, arterial partial pressure of carbon dioxide; *P*ao_2_, arterial partial pressure of oxygen; RV, right ventricle; *S*po_2_, peripheral oxygen saturation; TAPSE, tricuspid annular plane systolic excursion.

	Normoxaemia (*F*io_2_=0.3)	Hyperoxia (*F*io_2_=0.8)	*P*-value
*Intraoperative measures*			
Effective *F*io_2_	0.30 (0.05)	0.80 (0.01)	**<0.01∗**
ORi (0–1)	0.00 [0.00–0.23]	0.54 [0.18–1.00]	**<0.01∗**
*S*po_2_ (%)	96 (1)	99 (1)	**<0.01∗**
*P*ao_2_ (kPa)	12.7 (2.0)	37.5 (8.5)	**<0.01∗**
*P*aco_2_ (kPa)	5.2 (0.6)	5.2 (0.6)	0.98
Systolic blood pressure (mm Hg)	105 (13)	106 (12)	0.41
Mean arterial pressure (mm Hg)	72 (9)	72 (9)	0.21
Diastolic blood pressure (mm Hg)	56 (7)	56 (7)	0.47
Heart rate (beats min^−1^)	55 (10)	52 (8)	**<0.01∗**
*Standard TOE variables*			
TAPSE (mm)	23.7 (6.6)	23.0 (5.7)	0.19
Mitral valve inflow peak *E* (cm s^−1^)	62 (15)	65 (16)	**0.04∗**
Mitral valve inflow peak *A* (cm s^−1^)	50 (17)	48 (15)	**0.04∗**
Septal *e*′ (cm s^−1^)	7.0 (1.5)	6.9 (1.4)	0.26
Lateral *e*′ (cm s^−1^)	8.7 (2.0)	8.6 (1.8)	0.34
LVOT VTI (cm)	18.5 (4.5)	17.8 (4.4)	0.07
LVOT VTI volume (ml)	62 (18)	60 (18)	0.08
*E*/*A*	1.3 (0.4)	1.4 (0.4)	**0.01∗**
Septal *E*/*e*′	9.2 (2.5)	9.7 (2.8)	**0.01∗**
Lateral *E*/*e*′	7.6 (2.7)	7.9 (2.6)	0.09
Averaged *E/e*′	8.2 (2.4)	8.6 (2.6)	**0.01∗**
*3D LV function*			
End diastolic volume index (ml m^−2^)	57 (19)	57 (17)	0.66
End systolic volume index (ml m^−2^)	29 (14)	30 (13)	0.94
Stroke volume index (ml m^−2^)	28 (10)	27 (7)	0.28
Ejection fraction (%)	50 (10)	49 (9)	0.17
Cardiac output (L min^−1^)	3.7 (1.7)	3.8 (1.1)	0.30
Cardiac index (L min^−1^ m^−2^)	1.9 (0.9)	1.9 (0.6)	0.36
*3D LV longitudinal strain*			
Peak strain, GLS (%)	–15.7 (3.9)	–15.3 (3.1)	0.32
End systolic strain (%)	–14.4 (4.0)	–14.1 (3.1)	0.19
Time to peak strain (ms)	391 (66)	385 (81)	0.87
Mechanical dispersion (ms)	61 (36)	60 (34)	0.18
*3D RV function*			
End diastolic volume index (ml m^−2^)	71 (16)	70 (15)	0.43
End systolic volume index (ml m^−2^)	38 (10)	38 (10)	0.99
Ejection fraction (%)	46 (6)	45 (8)	0.51
Derived fractional area change (%)	33 (7)	33 (67)	0.82
*3D RV longitudinal strain*			
Free wall peak strain, RV-GLS (%)	–22.4 (4.8)	–21.9 (4.3)	0.35
Septal peak strain (%)	–13.4 (4.2)	–13.0 (3.8)	0.36

### Individual LV response to hyperoxia

**Fig 2:**
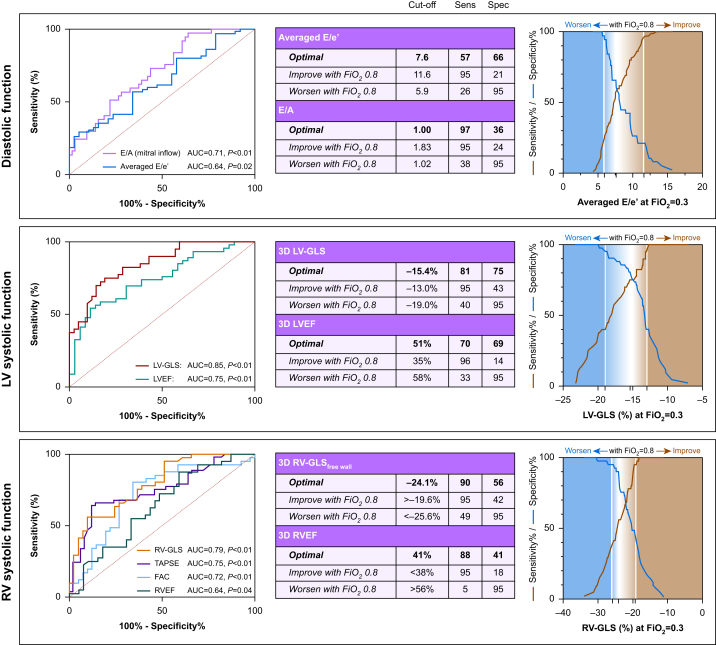
Left ventricular function. Left: receiver operating curves demonstrate the ability of 2D and 3D echocardiography measurements at normoxaemia (*F*io_2_=0.3) to discriminate patients who worsen in the respective parameter at hyperoxia (*F*io_2_=0.8) defined as an increase in *E*/*A*, *E*/*e*′, and GLS or reduction in EF, FAC, and TAPSE. Centre: optimal cut-offs from these curves determined by the Youden index are shown along with the cut-offs for the highlighted imaging variables that have a 95% specificity for detecting the patients who deteriorate with hyperoxia, along with 95% sensitivity cut-offs for detecting the patients who will improve with hyperoxia defined as a reduction in *E*/*A*, *E*/*e*′, and GLS or an increase in EF, FAC, and TAPSE. Right: sensitivity and specificity for *E*/*e*′ averaged for the septal and lateral wall, LV-GLS and RV-GLS are plotted based on normoxaemic values. ∗*P*<0.05 between blood gas levels. *F*io_2_, inspired oxygen fraction; EF, ejection fraction; FAC, fractional area change; GLS, global longitudinal peak strain; LV, left ventricular; RV, right ventricular; AUC, area under the curve.

**Fig 3:**
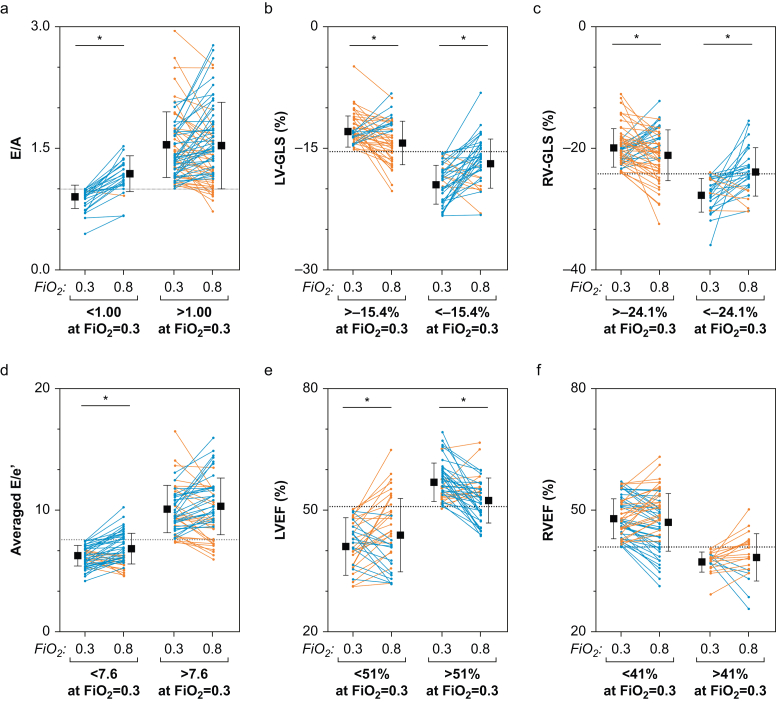
Response to hyperoxia based on functional status at normoxaemia. The response of echocardiographic measures to hyperoxia are grouped by the optimal cut-off of functional status at normoxaemia determined in Figure 2. Blue indicates a worsening with hyperoxia, and orange represents an improvement. ∗*P*<0.05 between blood gas levels. *F*io_2_, inspired oxygen fraction; EF, ejection fraction; GLS, global longitudinal peak strain.

ROC analysis was performed for LV systolic and diastolic function to investigate if LV systolic or diastolic function at normoxaemia can predict the myocardial response to hyperoxia. Systolic markers, LV-GLS (AUC=0.85, *P*<0.01) and LVEF (AUC=0.75, *P*<0.01; Fig 2), measured at normoxaemia discriminated patients who experienced worsening in LV systolic myocardial function when exposed to hyperoxia. The derived optimal cut-off value for LV-GLS was –15.4% (sensitivity and specificity provided in Fig 2). When applying the optimal LV-GLS cut-off to the study population, patients with a normoxaemic LV-GLS worse than the cut-off (more positive GLS) significantly improved with hyperoxia (–12.7% [1.9%] *vs* –14.1% [2.7%], *P*<0.01). In the subgroup with a baseline LV-GLS better than the cut-off of –15.4%, a significant worsening in LV-GLS was observed with transition to hyperoxia from –19.2 (2.4) to –16.6 (3.0), *P*<0.01 (Fig 3). Additional cut-offs derived by 95% sensitivity and specificity indicated that a stricter cut-off of –13.0% yields a high probability that ventricular function will benefit from hyperoxia, whereas hyperoxia has a high probability for inducing ventricular dysfunction in patients with LV-GLS better than –19.0%. With LVEF an optimal cut-off of 51% was calculated, and trends were similar to those of LV-GLS.

However, for diastolic markers there was only a moderate predictive ability of normoxaemic measures to discriminate diastolic function changes triggered by hyperoxia (*E*/*A*: AUC=0.71, *P*<0.01, *E*/*e*′: AUC=0.64, *P*=0.02). Optimal cut-offs were 1.0 for *E*/*A* and 7.6 for *E*/*e*′. When applying these cut-offs to *E*/*A* and *E*/*e*′, patients with diastolic markers under this cut-off (better diastolic function) worsened with hyperoxia. Although, unlike systolic markers, patients on the other side of the optimal cut-off (worse diastolic function) did not significantly benefit from hyperoxia (Fig 2).

### Individual RV response to hyperoxia

A heterogenous response was also observed regarding RV-GLS and RVEF. However, only hyperoxia-induced RV-GLS changes showed a significant relation to normoxaemic RV-GLS (*r*=–0.63, *P*<0.01). ROC curve analysis implied that free-wall RV-GLS can discriminate patients with reduced myocardial function under hyperoxia. The optimal cut-off value for RV-GLS was –24.1%. ROC curves are also provided for TAPSE and RV FAC in Figure 3, and cut-off values can be seen in Supplementary Table S3). Using this cut-off to the study population resulted in significant improvement with hyperoxia (–19.8% [3.2%] *vs* –21.0% [4.2%], *P*<0.05) in those with worse function than the cut-off. Whereas those with a normoxaemic RV-GLS better (more negative) than the cut-off had a significant decrease from –27.6 (2.8) to –23.8 (4.0)%, *P*<0.01 (Fig 3).

### Clinical safety outcomes

During the hyperoxic study period, only one patient exhibited depressed contractility of inferior wall segments, two showed new ECG changes typical for ischaemia, and two had P wave and A-wave loss in ECG and transmitral PW Doppler interrogation.

## Discussion

In this randomised cross-over trial involving 103 patients undergoing general anaesthesia before incision for CABG surgery, we found that diastolic function was consistently worse at hyperoxia. Biventricular systolic myocardial function did not significantly differ between normoxaemia and hyperoxia as determined by strain analysis and 3D volumetry. However, when relating individual hyperoxia-induced changes of systolic function to the normoxaemic state, we observed that patients with better myocardial function deteriorated during hyperoxia, whereas patients with poor function at normoxaemia improved at a higher *F*io_2_.

**Fig 4:**
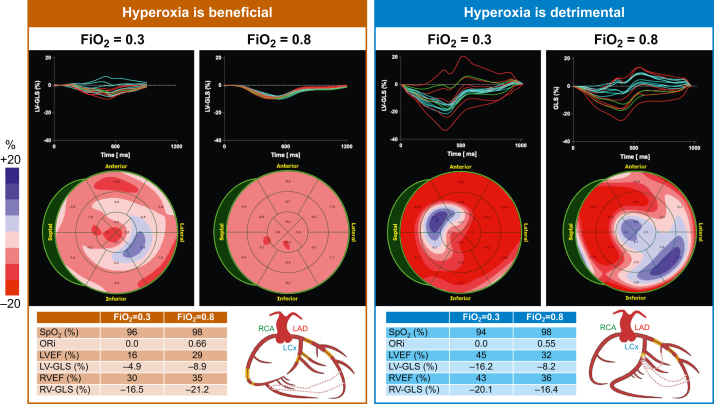
Case examples. Case examples demonstrate that (left) in a patient with three vessel disease and low ejection fraction and longitudinal strain at normoxaemia (*F*io_2_=0.3), hyperoxia (*F*io_2_=0.8) improves biventricular function and homogenises the regional strain response. On the other hand, in the patient with a stenosis of the left anterior descending coronary artery and occlusion of the circumflex (right), although there are some regional strain abnormalities at *F*io_2_=0.3, biventricular strain worsens with significant dysfunction arising in the circumflex territories in the lateral wall. In the longitudinal strain curves, red depicts the segments perfused by the left anterior descending coronary artery (LAD), whereas blue depicts the segments perfused by the left circumflex artery (LCx), and green by the right coronary artery (RCA). EF, ejection fraction; *F*io_2_, inspired oxygen fraction; GLS, global longitudinal peak strain; LV, left ventricle; ORi, oxygen reserve index; RV, right ventricle; *S*po_2_, peripheral oxygen saturation.

To our knowledge, this is the first study to show that patients with stable coronary artery disease under general anaesthesia show consistent worsening of diastolic function in response to hyperoxia. Diastolic dysfunction was present at both hyperoxia and normoxaemia with mean septal and lateral tissues velocity *e*′ under 8 and 10 cm s^−1^, respectively (Table 2). Guidelines for the assessment of diastolic function state that in the presence of reduced tissue velocities identifying diastolic dysfunction, an increase in *E*/*A* and *E*/*e*′ signifies a worsening of diastolic function.^19^ Diastolic dysfunction is an early feature in the myocardial ischaemic cascade and occurs before systolic contractile deficits.^20^ Worsening of cardiac output during hyperoxia has been described before; our results can attribute this phenomenon to depressed myocardial function in our population with coronary artery disease under general anaesthesia.^21^ ^22^ An explanation for such deterioration in diastolic and systolic function may be found in the well-described phenomenon of hyperoxia-induced coronary vasoconstriction. Such a response can lead to global maldistribution or complex inter-coronary redistribution of coronary blood flow in coronary artery disease patients with fixed stenoses. Importantly, under moderately hyperoxic conditions as in our protocol, a marginally enhanced blood oxygen content will be more than negated in such patients by a significant reduction of coronary blood flow. The resultant oxygen demand–supply mismatch may induce mechanical dysfunction of the subtended myocardium.^10^ Our results could thus be cautiously interpreted as a very early and sensitive marker for a shift of the delicate myocardial oxygenation balance towards deoxygenation. The consistent worsening of pre-existing diastolic dysfunction and deterioration of systolic function in select patients may be the sequential downstream functional sequalae of such an unfavourably shifted oxygenation balance.^14^^,^^23^^,^^24^ On the other hand, our results also indicate that patients with quite poor baseline function may demonstrate an improved contractility with transition to hyperoxia (Fig 4). With progressive coronary artery disease the probability of microvascular dysfunction in the coronary system also increases, and this could be a reason why patients with a higher ischaemic burden and lower systolic function at normoxaemia may benefit from hyperoxia.^25^ Our data enabled us to determine cut-off values for systolic variables derived from 3D datasets to discriminate patients for whom hyperoxia is disadvantageous from those who benefit from it. This effect has also not been explicitly described yet.

In predicting myocardial performance under hyperoxia, strain analysis had the highest AUC for both ventricles. Myocardial strain is a sensitive marker for myocardial dysfunction in coronary artery disease and enables prognostication of adverse cardiac events. In this regard it performs better than ejection fraction, and is therefore included in recent recommendations for echocardiographic chamber quantification.^15^^,^^26^^,^^27^ Moreover it can quantify dynamic changes in function occurring over a short period.^28^, ^29^, ^30^, ^31^ As yet, under the conditions of a cardiac operating room, this type of analysis appears relatively time consuming, but will eventually benefit from further automatisation. As conventional and much quicker analysis variables, TAPSE and FAC also showed limited utility in predicting RV response to hyperoxia.

### Clinical implication of findings

To preserve diastolic function, a normoxaemic *F*io_2_ may be more favourable in stable coronary artery disease patients undergoing general anaesthesia. Our results, however, indicate that selecting the *F*io_2_ during general anaesthesia can have a perceptible impact on systolic myocardial performance in patients with coronary artery disease. The results of our (non-stratified) analysis are in accordance with a recent prospective study, which also did not find any general benefit of normoxaemia *vs* hyperoxia during general anaesthesia. However, stratifying patients at risk according to their staged response to *F*io_2_ changes, as suggested in our *post hoc* analysis, may result in tailored management of oxygenation during general anaesthesia and thus benefit cardiac performance in patients with established coronary artery disease.^22^^,^^32^ A recent large observational cohort study showed that raising *F*io_2_ was associated with increased odds of myocardial injury until 30 days after noncardiac surgery.^7^ This association was, however, not confirmed by a controlled trial, and the authors assumed unobserved confounders in observational datasets as an explanation.^7^ ^33^ Nevertheless, our results suggest that the *F*io_2_ setting in individuals with vulnerable coronary anatomy and function may also affect clinical cardiac outcomes. This study, however, only investigates short-term changes in function as the outcome: future analysis will need to investigate independent outcomes such as cardiac biomarkers and data on morbidity and mortality. Preoperative evaluation of myocardial function with strain analysis and testing for changes in GLS in response to defined hyperoxic and hypocapnic challenges may help to adapt and improve intraoperative management of oxygenation and ventilation.^14^^,^^28^ Given our finding of a highly individual response to hyperoxia, such personalised management appears worthy of further studies into improvement of clinical outcome.

### Limitations

In our study, TAPSE, mitral valve inflow and tissue Doppler variables were prone to acquisition and observer bias as they were acquired and initially analysed by a non-blinded echocardiographer. However, a *post hoc* blinded analysis of mitral inflow variables and tissue Doppler in 20 random samples showed excellent reliability with the non-blinded *ad hoc* primary analysis (ICC) (Supplementary Table S2). The TOMTEC analysis modules for 3D data did not support analysis of TAPSE and diastolic strain rate. A planned future analysis of the 2D datasets of the study population may provide further insight into changes of LV and RV diastolic function during changes of *F*io_2_. There was a large standard deviation in myocardial markers at each level arising from between-patient variance at both oxygenation levels, and this is attributable to the wide range in function between individuals. These, however, do not represent the within-patient variation of the individual changes when transitioning from normoxaemia to hyperoxia. Nevertheless, differences in means between oxygenation levels were small, yet still statistically significant when investigating diastolic function. Stratifying the patients by the derived cut-offs for systolic and even diastolic function highlights the individual responses suggesting an individualised approach could be ideal. Our LV-GLS cut-off results derived under general anaesthesia conditions were already in a range that can be classified as diminished strain. Despite its technical and clinical feasibility there are still few research groups which utilise strain analysis perioperatively, even more so at TOE and on 3D datasets. Strain analysis results not only depend on the modality but also on the vendor of the hardware and software used. When compared with transthoracic echocardiography, TOE-based strain analysis reportedly tends to overestimation. Also, 3D echocardiography yields larger chamber volumes than 2D scans. Therefore, external validity of our strain data and its comparability with data from the scarce literature is currently still limited.^17^^,^^34^^,^^35^

## Conclusion

This randomised cross-over clinical trial in anaesthetised patients with well-defined stable coronary artery disease before incision for elective CABG surgery found a significant worsening of diastolic function at hyperoxia but no significant changes in systolic function parameters. Individually, hyperoxia yielded very heterogeneous effects on systolic myocardial function. coronary artery disease patients with impaired contractile function at normoxaemia improved, whereas myocardial contractility of patients with better systolic function at normoxaemia deteriorated with hyperoxia in both ventricles. Perspectives of this research are to preoperatively assess predictors for the perioperative hyperoxic myocardial response to personalise intraoperative oxygen management for high-risk coronary artery disease patients, and to relate such findings to clinical cardiac outcomes.

## Authors' contribution

Acquisition of data: JOF, JM, RB, RR, KF, AL, SB, TH, DG, GE, BE, DPG.

Analysis of data: JOF, JM, RB, RR, KF, AL, SB, TH, DG, GE, RM, BE, DPG.

Interpretation of data: all authors.

Drafting of the article: JOF, KF.

Study administration: KF, DPG.

Conception and design of the study: KF, DPG, BE.

Critical revision for important intellectual content: KF, DG, GE, FSS, TPC, RM, BE, DPG.

Final approval of the version to be submitted: DPG.

All authors agree to the final version of this manuscript and are accountable for all aspects of the work in ensuring that questions related to the accuracy or integrity of any part of the work are appropriately investigated and resolved.
