# Supplementary material for: Hyperoxia-induced deterioration of diastolic function in anaesthetised patients with coronary artery disease – Randomised crossover trial
Source: BJA Open. 2023 Apr 27;6:100135. doi: 10.1016/j.bjao.2023.100135 (PMC10430862; doi:10.1016/j.bjao.2023.100135)
Supplement: Multimedia component 1 [file mmc1.docx]

SUPPLEMENTAL MATERIAL

**Hyperoxia-induced deterioration of diastolic function in anaesthetized patients with coronary artery disease – A randomized crossover trial**

Jan O. Friess^1,2^; Jan Mikasi^1^; Rico Baumann^1^; Rajevan Ranjan^1^; Kady Fischer^1^; Anja Levis^1^; Sandra Terbeck^1^; Trevor Hirschi^1^; Daniel Gerber^1^; Gabor Erdoes^1^; Florian S. Schoenhoff^3^; Thierry P. Carrel^3^; Raouf Madhkour^4^; Balthasar Eberle^1^; Dominik P. Guensch^1^

1. Department of Anaesthesiology and Pain Medicine, Inselspital, Bern University Hospital, University of Bern, Bern, Switzerland.
2. Department of Anesthesiology, Critical Care and Pain Medicine, Boston Children's Hospital, Harvard Medical School, Boston, United States.
3. Department of Cardiovascular Surgery, Inselspital, Bern University Hospital, University of Bern, Bern, Switzerland.
4. Department of Cardiology, Inselspital, Bern University Hospital, University of Bern, Bern, Switzerland.

[Supplemental Methods: 2](#_Toc125472162)

[Supplemental Figure I: Consort Figure 4](#_Toc125472163)

[Supplemental Table I: Additional Echocardiographic Parameters 5](#_Toc125472164)

[Supplemental Table 2: Reliability Analysis 6](#_Toc125472165)

[Supplemental Table 3: Additional Right Ventricular Optimal Cut-offs 7](#_Toc125472166)

# Supplemental Methods:

*Detailed patient inclusion and exclusion criteria*

Patients were included if they were 1) undergoing elective coronary artery bypass graft (CABG) surgery, 2) older than 18 years of age, and 3) able to provide informed consent. Exclusion criteria included absolute contraindication for TEE, patients where study explanation and informed consent cannot be performed/obtained at the latest on the day before scheduled surgery, emergency surgery, patients with unstable CAD: ST- and Non-ST-elevation myocardial infarction and unstable angina. Further exclusion criteria were atrial fibrillation or significant arrhythmia, pacemakers, cardiac resynchronization therapy, left bundle branch block, severe-grade valvular disease, pericardial disease, previous cardiac or thoracic aortic surgery, chest radiation therapy, cardiotoxic or bleomycine chemotherapy, severe pulmonary hypertension, cor pulmonale, or right ventricular dysfunction and finally females of child-bearing potential.

*Image Analysis*

For 3D analysis of the left ventricle (LV), the coded 3D datasets were loaded into the “4D-LV Analysis”-module of TOMTEC-Arena (Build No. 514944). In the first step the LV extent was defined, i.e., extent above the mitral valve on the atrial side to the apex of the heart. Next, the central axis was rotated in order to match the 4-chamber, 2-chamber and 3-chamber views in the correct order provided in the pictogram on the right side of the module. After this step, the end-diastolic and end-systolic frames were auto-detected by the electrocardiogram trace of the 3D acquisition. This automated process was confirmed by the reader and if required adjusted by checking the maximal and minimal ventricular volumes and closure of the valves. In the next analysis step, the program automatically traced the endocardial border in the 4-, 2- and 3-chamber views, the mitral valve level and aortic valve. If required, the reader re-adjusted the contours in the long axis and short axis views within the end-diastolic and end-systolic frames and checked throughout the cardiac cycle for correct tracing by reviewing the cine. If tracing could not be corrected the dataset was excluded from analysis. In the case of good tracing, the analysis button was activated, and a volume and strain curves for the LV were displayed together with numerical results. These were exported for statistical analysis.

The 3D analysis of the right ventricle (RV) was performed in the “4D RV-Function” module. In the first step a pictogram with the required orientations were provided on the right side of the screen. Initially, the reader manually defined the LV extent, and then secondly defined the RV extent in two orthogonal views which extended from above the tricuspid valve to the apex. Correct short axis orientation was confirmed on the right side of the screen. As the next step the “tracking revision” button was activated. While endocardial borders were automatically detected, endocardial contours needed to be defined in the long and short axis views due to the complex shape of the RV, i.e., the short axis planes had to be moved along the long axis centre and the long axis views were rotated by adjusted the cutting angle in the short axis views for good visualisation and tracking revision in the end-diastolic and -systolic frames. Correct tracking was confirmed throughout the cardiac cycle and further adjusted, if necessary. In addition, in the tricuspid valve view, the detected septal and lateral valve border could be re-adjusted for correct volumetry, as well as for the pulmonary valve. Readers could also verify the 3D model generated from the contours for accuracy. If tracing was deemed correct, the numeric global results, as well as a volumetric curve were displayed after hitting the analysis button. RV diameters, fractional area change (FAC) and septal and free wall RV longitudinal strain results were displayed. By clicking on FAC and strain parameters the autodetected myocardial borders were displayed and could be corrected if required. Tricuspid annular plane systolic excursion (TAPSE) measurements were not displayed, as they are disabled if the software recognizes an image orientation in accordance with transoesophageal images. TAPSE is currently only available for 3D datasets acquired by transthoracic echocardiography. Results were exported for further analysis.

*Supplemental Statistical Methods*

For the secondary analysis, receiver operating characteristic (ROC) curves were used to calculate the ability of normoxaemic imaging results to discriminate if individual patients benefited or not in the individual echocardiographic parameter with hyperoxia. For this analysis the outcome was a binary variable categorized as worsening with the transition from normoxemia to hyperoxia, or as improvement in the individual parameter with a higher FiO_2_. For each parameter (i.e., E/A, LVEF), the outcome was the worsening or improving of the individual parameter. For example, if LVEF dropped from 55% at normoxaemia to 45% at hyperoxia, the outcome was categorized as “worsening”. In all cases the threshold was set at a delta change of 0. The functional measurements at normoxaemia were input as a continuous variable into the ROC analysis. The ROC curve then plotted sensitivity to 1-specificity for all possible threshold values of normoxaemia measurements to discriminate the outcome. An optimal cut-off point was derived using the Youden index, which finds the point with the best combination of sensitivity and specificity.

An example of how an ROC analysis determines the true positives (TP), true negatives (TN), false positive (FP) and false negatives (FN) for each decision threshold is shown in the table below using the LV-GLS of five patients. From these values a true positive rate (TP/(TP+FN)) and false positive rate (FP/(FP+TN)) is calculated to form the ROC curve. For the example, we have chosen three points on the curve, the optimal cut-off point (Youden index), and the points when sensitivity and specificity are 95%. Nevertheless, an ROC performs this type of analysis for each point shown on the curve, thereby also generating the area under the curve (AUC) value. The AUC can be interpreted as the probability that the normoxaemic measurement of the group that worsens with FiO_2_ of 0.8 was significantly different than the normoxaemic measurement the group that improved. Further analysis will need to validate these cut-offs with an external cohort with contingency tables. Contingency tables were not a part of this analysis.

|  | **Measured Values** | | **ROC Derived Outcome** | | |
| --- | --- | --- | --- | --- | --- |
| **Patient** | **LV-GLS at FiO_2_=0.3** | **Outcome**  1: LV-GLS worsened with FiO_2_=0.8 (>Δ0%)  0: LV-GLS improved with FiO_2_ 0.8 (<Δ0%) | **Point 1: Orange**  95% Sensitivity  Threshold: -13.0% | **Point 2: Purple**  Optimal  Threshold: -15.4% | **Point 3: Blue**  95% Specificity  Threshold: -19.0% |
| 1 | -13.8% | 0 | 1 (FP) | 0 (TN) | 0 (TN) |
| 2 | -21.5% | 1 | 1 (TP) | 1 (TP) | 1 (TP) |
| 3 | -4.9% | 0 | 0 (TN) | 0 (TN) | 0 (TN) |
| 4 | -18.5% | 1 | 1 (TP) | 1 (TP) | 0 (FN) |
| 5 | -15.6% | 1 | 1 (TP) | 1 (TP) | 0 (FN) |

TN: True negative when the ROC curve accurately defines a patient will improve with FiO_2_=0.8 based on the given threshold of LV-GLS at FiO_2_=0.3 and the patients measured results show they had improved (reference outcome).

FN: False negative when the ROC curve incorrectly defines a patient will improve with FiO_2_=0.8 based on the given threshold of LV-GLS at FiO_2_=0.3 but the patients measured results show they had worsened (reference outcome).

TP: True positive when the ROC curve accurately defines a patient will worsen with FiO_2_=0.8 based on the given threshold of LV-GLS at FiO_2_=0.3 and the patients measured results show they had worsened (reference outcome).

FP: False positive when the ROC curve incorrectly defines a patient will worsen with FiO_2_=0.8 based on the given threshold of LV-GLS at FiO_2_=0.3 but the patients measured results show they had improved (reference outcome).

Janssens ACJW, Martens FK. Reflection on modern methods: Revisiting the area under the ROC Curve. *Int J Epidemiol* 2020; **49**: 1397–403

# Supplemental Figure I: Consort Figure


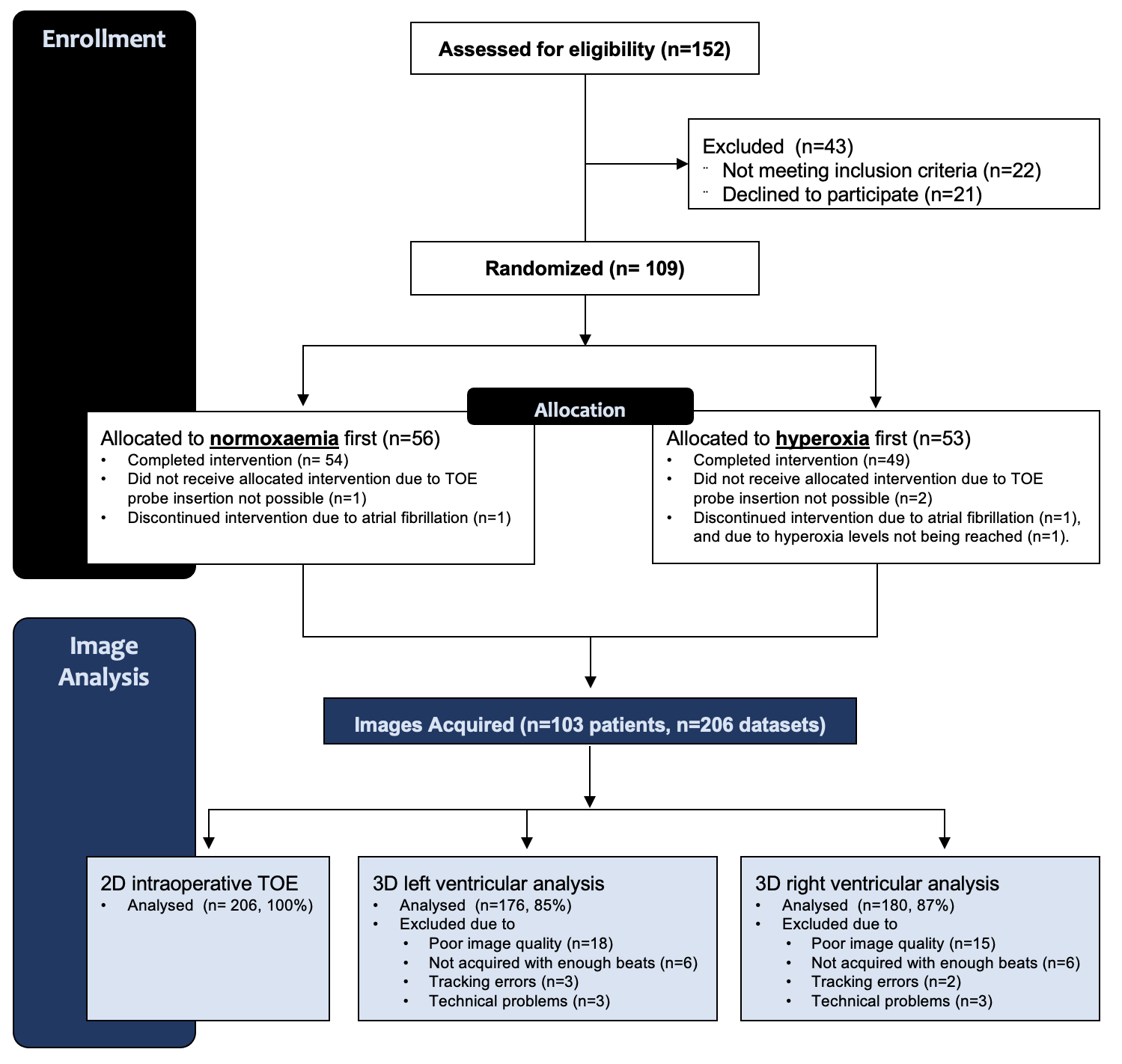


Of 109 patients randomized, 103 completed the study. In total 206 datasets from the two gas levels of 103 patients were included in the final analysis. D: Dimension, TOE: transoesophageal echocardiography.

# Supplemental Table I: Additional Echocardiographic Parameters

|  | **Normoxaemia** | **Hyperoxia** | **p-value** |
| --- | --- | --- | --- |
| *3D LV Function* |  |  |  |
| End Diastolic Volume (ml) | 114 (34) | 115±38 | 0.64 |
| End Systolic Volume (ml) | 59 (27) | 60 (26) | 0.92 |
| Stroke Volume (ml) | 56 (21) | 54 (13) | 0.22 |
| *3D LV Circumferential Strain* |  |  |  |
| Peak Strain (%) | -25.7 (7.9) | -24.8 (7.0) | 0.17 |
| End-Systolic Strain (%) | -21.7 (6.7) | -21.2 (6.2) | 0.46 |
| Time to Peak Strain (ms) | 351 (73) | 349 (71) | 0.76 |
| Mechanical Dispersion (ms) | 69 (21) | 65 (16) | 0.05 |
| *3D RV Function* |  |  |  |
| End Diastolic Volume (ml) | 144 (36) | 143 (35) | 0.80 |
| End Systolic Volume (ml) | 79 (23) | 79 (24) | 0.85 |
| Stroke Volume (ml) | 65 (17) | 64 (18) | 0.59 |

Mean±standard deviation is displayed for measurements obtained at each level. For all parameters, there was no significance in the order that the levels were performed. LV: left ventricle, RV: right ventricle.

# Supplemental Table 2: Reliability Analysis

|  | **ICC** | **p** |
| --- | --- | --- |
| 3D LV Peak Global Longitudinal Strain | 0.80 | <0.01 |
| Mitral Valve Inflow Peak E (cm s^-1^) | 0.96 | <0.01 |
| Mitral Valve Inflow Peak A (cm s^-1^) | 0.99 | <0.01 |
| E/A | 0.98 | <0.01 |
| Septal e’ (cm s^-1^) | 0.99 | <0.01 |
| Lateral e’ (cm s^-1^) | 0.94 | <0.01 |
| Averaged E/e’ | 0.95 | <0.01 |

Intraclass correlation coefficients (ICC) are shown for inter-reader reliability of 20 datasets. LV: left ventricular.

# Supplemental Table 3: Additional Right Ventricular Optimal Cut-offs

|  | **Cut-off (N_0.3_)** | **Sensitivity** | **Specificity** |
| --- | --- | --- | --- |
| *RV Fractional Area Change (FAC)* |  |  |  |
| **Optimal** | **32%** | **80** | **66** |
| Improve with FiO_2_ 0.8 | <23% | 95 | 7 |
| Worsen with FiO_2_ 0.8 | >43% | 12 | 95 |
| *TAPSE* |  |  |  |
| **Optimal** | **25mm** | **64** | **88** |
| Improve with FiO_2_ 0.8 | <17mm | 94 | 22 |
| Worsen with FiO_2_ 0.8 | >30mm | 25 | 94 |

Optimal cut-offs based on the measurement at normoxaemia (N) for determining which patients worsen with right ventricular (RV) function when the fraction of inspired oxygen is titrated from 0.3 to 0.8. TAPSE: tricuspid annular plane systolic excursion.
